# Cost utility analysis of cryopreserved amniotic membrane versus topical cyclosporine for the treatment of moderate to severe dry eye syndrome

**DOI:** 10.1186/s12962-020-00252-6

**Published:** 2020-12-01

**Authors:** Jeffrey Voigt

**Affiliations:** Ridgewood, NJ USA

**Keywords:** Cyclosporine, Amniotic membrane, Dry eye syndromes, Analysis cost utility

## Abstract

**Background:**

The purpose is to perform a cost effectiveness analysis amniotic membrane vs. topical medications in the use of treating dry eye disease. A cost effectiveness analysis comparing amniotic membrane + other topical medications to topical cyclosporine A + other topical medications was evaluated using accepted decision tree modeling software.

**Methods:**

TreeAge Pro 2019 software was used to evaluate the base case costs over a one year timeframe. Sensitivity analysis was performed on those variables which had the greatest effect on choosing one therapy versus the other based on cost. Monte Carlo simulation was run 1,000 times to determine the most effective, least costly alternative. Costs were evaluated from a societal level (direct + indirect). Quality of life utility scores were evaluated using known time tradeoffs from prior studies (scale 0–1; with 1 being perfect vision).

**Results:**

Over a one year timeframe, the base case demonstrated that amniotic membrane + topical medications was the less expensive alternative and provided for incremental utilities versus topical cyclosporine + other medications (Cost/utility: $18,275/0.78 vs. $20,740/0.74). If examining direct costs only, topical cyclosporine was the least expensive option over a one year timeframe: $4,112 vs. $10,300. Sensitivity analysis demonstrated that in order for topical cyclosporine to be the less expensive alternative the following variables would need to be: < 68 days productivity lost; < $161 productivity lost/day; > 79% of amniotic membrane implants would need to be re-implanted at month 4 (for whatever reason); > $2677 per amniotic membrane implant procedure (Medicare reimbursement rate); > 96% positive response to topical cyclosporine A at month 4; > 58% positive response to topical cyclosporine A at month 6 and; < 54% probability clinical improvement with amniotic membrane. Monte Carlo simulation demonstrated that amniotic membrane was the less costly, most effective alternative 91.5% of the time.

**Conclusion:**

Based on improved outcomes using amniotic membrane, patient productivity was improved resulting in lower societal costs (less days lost from work). When considering the untoward effects of dry eye disease on societal costs, an improvement of the dry eye disease condition was accomplished most often with amniotic membrane.

## Introduction

Dry eye as a disease (DED) (keratoconjunctivitis sicca) is prevalent in 5–50% of the population worldwide [1]. In the United States DED is one of the most frequently identified ocular morbidities with over 4 million people over 65 DED symptomology [2]. The costs incurred from the condition relate not only to the direct costs for care in treating DED but as well indirect costs—most especially, productivity loss due to work absences or hours of reduced effectiveness at work—resulting from DED sequelae. Direct costs from a payer perspective for treating DED in the US are estimated at $3.84 billion [3]. Indirect US societal costs incurred from DED are estimated at $55.4 billion [3]. In other developed countries, direct costs are estimated on a per person basis with: $530 ± $384 in Japan; $1,100 in United Kingdom; $273 in Germany, $645 in Italy, $765 in Spain, and $273 in France [3]. DED symptoms affect a person’s quality of life and include irritation, stinging, dryness, ocular fatigue, neurosensory dysfunction, and visual disturbances [4, 5]. These symptoms can result in localized and/or bodily pain, decreased role-physical, vitality and general health scores when measured via Quality of Life (QoL) instruments and; worsen as DED increases in severity [6].

DED as a condition can worsen over time and based on this, as signs and symptoms intensify, more frequent and different applications of topical medications can ensue. These topical medications can include: artificial tears, anti-inflammatories, and cyclosporine A. The therapeutic management of DED also typically involves addressing chronic sequelae.

Treatment for DED is based on its severity and chronicity. DED severity levels have been defined by an international task force along with recommended treatments for each severity level [7]. As DED becomes more severe, more invasive options (e.g. surgery) become an option to control the inflammatory effects of DED [7].

Amniotic membrane (AM) has been evaluated in severe high-risk ocular pathology (e.g. corneal ulcers, neurotrophic keratopathy) with moderate to reasonably good success [8, 9]. Recently treatment of DED has been evaluated using AM for treatment of more severe DED [10–12]. One of the more interesting findings in these studies is the effect of AM on corneal nerve regeneration (for relieving pain) along with accelerated recovery of the ocular surface in patient with DED [13]. Corneal nerve dysfunction/damage unfortunately is not routinely tested for (or treated) in the clinical setting [5].

To date there have been cost analyses on the use of topical medications over periods of time and based on symptomatology [14–16]. As well, cost utility analyses have been reported on using topical cyclosporine in treating dry eye vs. a sham [17]. However, to date a cost-utility analysis has not been undertaken comparing the use of topical cyclosporine (a common treatment) to AM in treating moderate to severe DED. It is the intention of this analysis to compare the use of AM vs. topical cyclosporine in the treatment of moderate to severe DED in this regard.

## Methods

Costs and utility were evaluated using Tree-Age Pro 2019 (Williamstown, MA) in patients with moderate to severe DED. Data from prior studies was used for AM outcomes and for cyclosporine A, FDA clinical trial data was used as described below. The patients included in the analysis presented with moderate to severe DED and are described below. Patients were fairly well matched based on sex, age, and DED diagnosis. Tree-Age Pro is a cost-utility modeling software program. The cost and utility [18] were evaluated over a one year timeframe based on the data available. Cyclosporine A + topical medications were used as the comparison group based on its widespread use in the United States [19]. A societal perspective (direct and indirect) for examining costs was used. Where appropriate, the incremental cost utility was analyzed using the following formula: incremental cost/incremental quality adjusted life year (QALY): (1 year costs AM + topical medications less 1 year costs cyclosporine A + topical medications/(Incremental QoL of AM at year 1 less incremental QoL of cyclosporine A at year 1). If one therapy was less costly and more effective, that therapy was said to dominate the other in cost-utility analysis. The Consolidated Health Economic Evaluation Reporting Standards (CHEERS) checklist was used (Additional file 1: Appendix S1).

### Treatment and utility

The utility of cyclosporine A ± topical medications was used based on the Restasis clinical results as reported in its Food and Drug Administration (FDA) phase III submission [20]. There were 877 patients enrolled in the Restasis trials with a mean age of 58.3–60.8 years (depending upon the arm of the trial with the vast majority female (80 + %); and 28.1–36.7% with Sjorgen syndrome. Additionally the treatment paradigm for use of cyclosporine A + topical medications in DED (based on severity) followed the international panel of ophthalmic experts [7]. The results as reported on in the FDA submission were used over a one year time frame.

The utility of AM + topical medications vs. cyclosporine A + topical medications in treating DED was derived from the literature. For the AM (PROKERA, TissueTech, Miami, Florida, USA), data was derived from studies identified in a systematic review and are shown in Table 1. Per PROKERA’s 510 K #K032104, it is intended for use in eyes in which ocular surface cells are damaged or underlying stroma is inflamed or scarred. The systematic review Preferred Reporting Items for Systematic Reviews and Meta-Analyses (PRISMA) diagram and methodology can be found Additional file2: Appendix S2. Based on the literature review it was assumed that patients who were treated with AM, were also treated aggressively with adjunctive topical medications. It was further assumed based on the success rates (lessening of the severity of DED), that AM was reinserted (or not) over 4 month intervals. Adjunctive topical medications were administered over the entire year and included (depending upon the severity treatment recommendations^7^): artificial tears, cyclosporine A, and anti-inflammatory medications.

**Table 1:** AM results in patients with moderate to severe DED:

Study	Ref	Study design	Number patients	Outcomes
Cheng AMS, et al. Ocul. Surg. 2016	[10]	Retrospective review of 15 eyes in patients with moderate to severe dry eye disease and refractory to maximal medical treatment. Demographics: 2 males/8 females; age: 68.7 ± 16.2 years	10	Patients symptom free at 4 months. However after 4 months, symptoms recurred including itchy eye, blurred vision, burning sensation, dryness
John T, et al. Jr. Ophthal. 2017	[11]	Randomized controlled trial 17 patients—CAM (9) or conventional maximal treatment (8)—with moderate to severe dry eye disease. Demographics: 4 males/13 females; age: 67.8 ± 8.9 years; all presenting with moderate to severe DED grades 2–4	17	Patients in CAM group—symptoms improved dramatically after 3 months with adjunctive topical meds as needed. Control group symptoms remained the same with maximal medical treatment
McDonald MB, et al. Clin. Ophthal. 2018	[12]	Retrospective review of 97 eyes in patients with severe dry eye disease refractory to maximal medical treatment. Demographics: 12 male/69 female; all with severe DED; 86% manifested with superficial punctate keratitis	84	Patients ocular surface stable after 3 months with continued use of conventional treatments including artificial tears (96%); Restasis (57%) and steroids (32%)
Morkin MI, et al. Ocul. Surf. 2018	[13]	Retrospective cases series of 9 patients with DED and acute neuropathic corneal pain. Demographics: 1 male/8 females; age: 58.8 ± 4.3 years	9	During follow up of 9.3 ± 0.8 months, pain severity improved significantly. As well, only 2 out of the 9 patients required re-implantation due to pain recurrence

For clinical efficacy (improvement or degradation), the treatments as identified by the panel of international experts by DED severity were employed [7]. The DED severity levels corresponded to QoL utility assessments reported on previously in patients with DED [2]. The utilities were based on a scale of 0–1, with 1 being perfect vision and 0 being blindness). These utilities were used in the cost-utility model [2]. The QoL utility assessments were evaluated every 4 months over the year’s timeframe based on the condition of the patient (i.e. probability patient would be in that condition at that point in time) and then averaged.

### Treatment and cost

#### Direct costs

For cyclosporine A + topical medications the costs for topical medications were derived from the National Average Drug Acquisition Cost (NADAC) for July 2019 [21]. If the DED severity worsened based on outcomes as identified in the FDA phase III submission as per above, other therapies were administered including surgery (e.g. punctal plugs) as per the international panel of ophthalmic experts [7]. Costs based on treatments by DED severity for topical medications were either added or subtracted based on the patient condition over the course of a year. Costs for surgery were based on 2019 national average Medicare payments for the appropriate CPT code.

For AM, treatment included surgery using appropriate CPT codes and Medicare payment for the non-facility setting + topical medications. Again depending upon the outcome, a second or third AM insertion procedure was performed and topical medications were used.

#### Indirect costs

The costs due to missed work or lower productivity, based on DED severity, were derived from survey data [15]. Missed days from work or lower productivity at work (in hours) were then multiplied by the current Bureau of Labor Statistics average hourly wage [22]. The assumption was that at baseline each patient had moderate DED and their condition either improved or worsened with a corresponding utility gain or loss [2].

The costs, utilities and probabilities can be found in Additional file 3: Appendix S3. Additional file 4: Appendix S4 shows the calculations used for each terminal node in the cost-utility model.

Sensitivity analysis of those variables which affected the model the most (i.e. a change in their value resulted in a lower cost of one treatment vs. the other) were identified and evaluated. Sensitivity analysis is used to examine the potential impact of parameter assumptions and other uncertainties. A tornado plot identified these variables and separate one way sensitivity graphs were generated. Further, cost-effectiveness was evaluated in Monte Carlo simulation (1,000 times) and graphed as an incremental cost utility scatterplot.

Statistics including costs, outcomes, and, sensitivity analysis were employed using those included in the Tree-Age Pro software program.

## Results

**Fig. 1:**
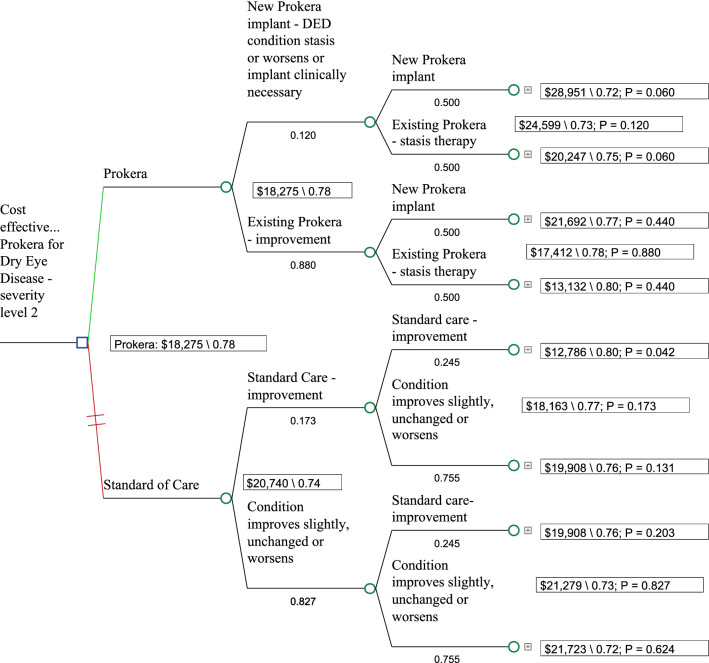
Decision tree: Prokera vs. Standard of Care (Cyclosporine A; Restasis)

**Fig. 2:**
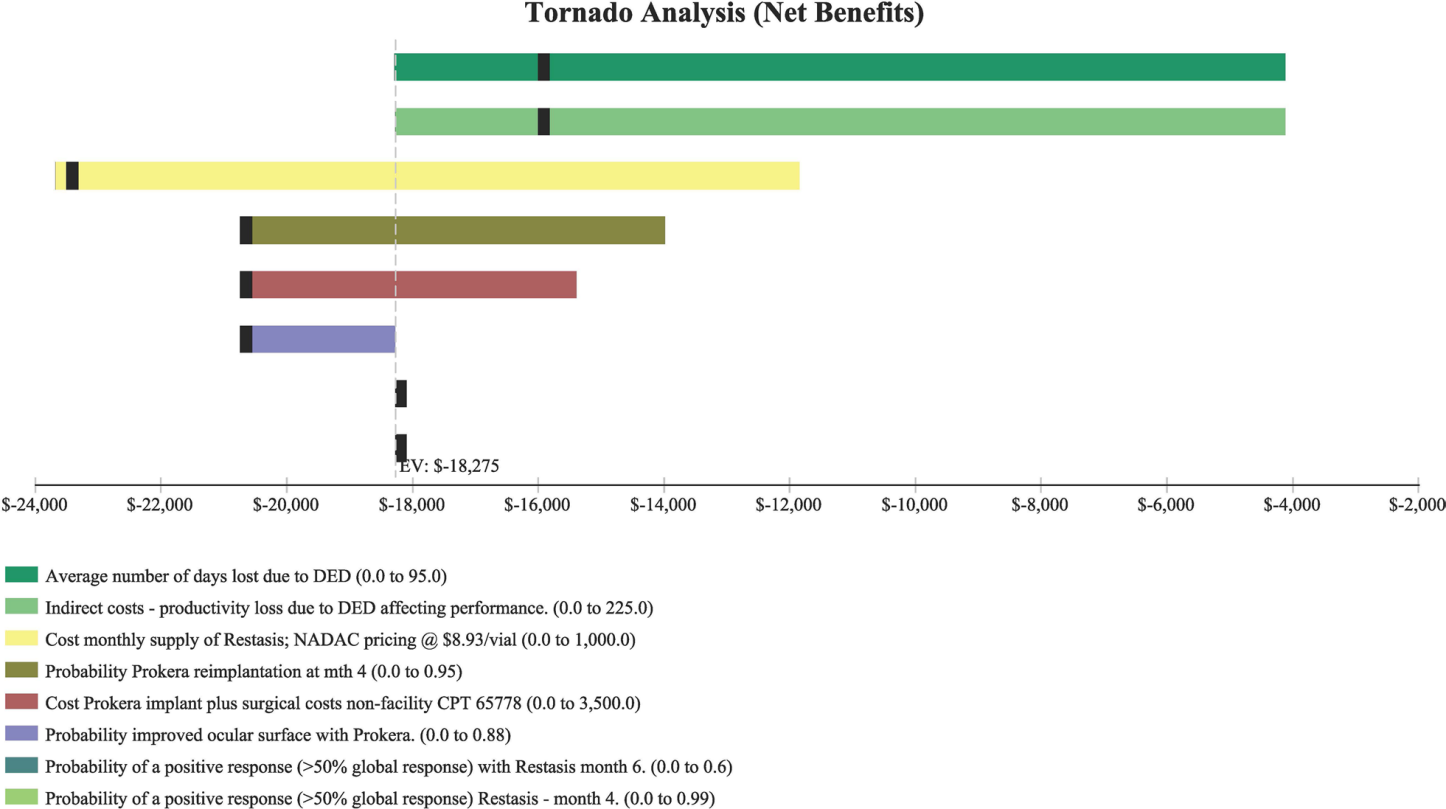
Tornado Diagram studying the impact of individual parameters/variables, that had the greatest impact on costs

Figure 1 shows the model and its base case results. As can be seen in the base case. Over a one year timeframe Fig. 2, AM + topical medications was the less expensive alternative and provided for incremental utilities versus topical cyclosporine + other medications (Cost/utility: $18,275/0.78 vs. $20,740/0.74).

Table 2 shows sensitivity analysis and demonstrated that in order for topical cyclosporine to be the less expensive alternative the following variables would need to be: < 68 days productivity lost; < $161 productivity lost/day; > 79% of AM insertions would need to be re-inserted at month 4 (for whatever reason); > $2,677 per AM insertion procedure (Medicare rate); > 96% positive response to topical cyclosporine at month 4; > 58% positive response to topical cyclosporine at month 6 and; < 54% positive response to AM.

**Table 2:** Sensitivity analysis

Variable	Base case used in model	Value at which cyclosporine A became the less expensive alternative	Figure
Productivity days lost	95 days	< 68 days	Fig. 3
Cost productivity per day	$225	< $161	Fig. 4
Monthly cost Restasis	$535	> $972	Fig. 5
Percent of time an AM insertion would need to be re-inserted at month 4	50%	> 79%	Fig. 6
Cost for an AM surgical implantation	$1,445	> $2,677	Fig. 7
Probability of clinical improvement with AM	88%	< 54%	Fig. 8
Probability positive response Restasis month 4	17.3%	> 96%	Fig. 9
Probability positive response Restasis month 6	24.5%	> 58%	Fig. 10

**Fig. 3:**
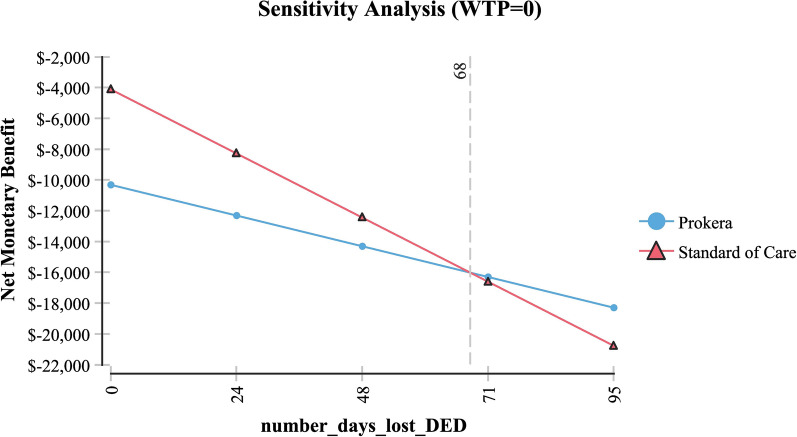
Sensitivity analysis number of work days lost due to dry eye disease showing that at <68 days lost, Cyclosporine A (Restasis) is the less expensive option for treatment

**Fig. 4:**
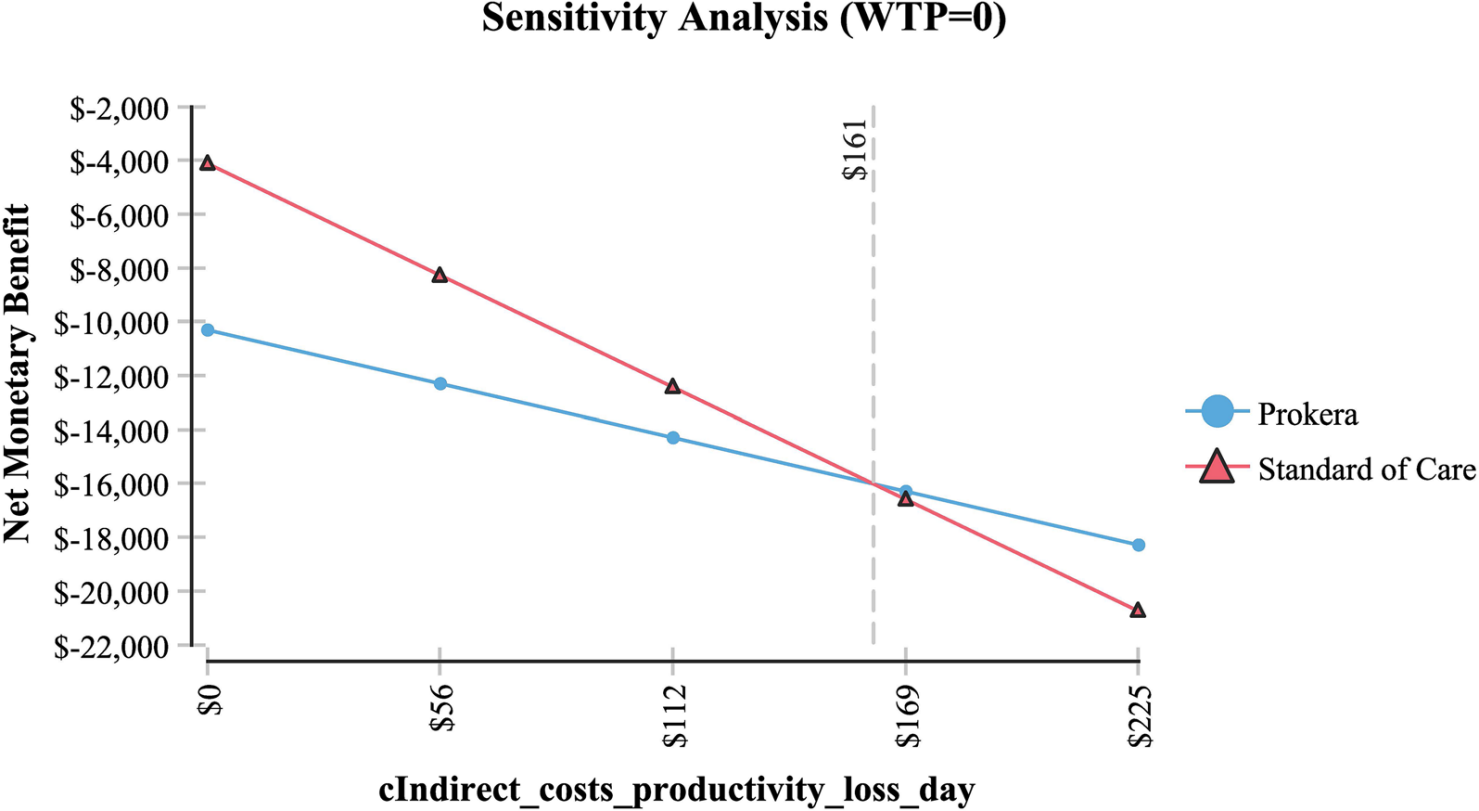
Sensitivity analysis showing that if productivity costs are < 161 day, then Cyclosporine A (Restasis) would be the less expensive option for treatment

**Fig. 5:**
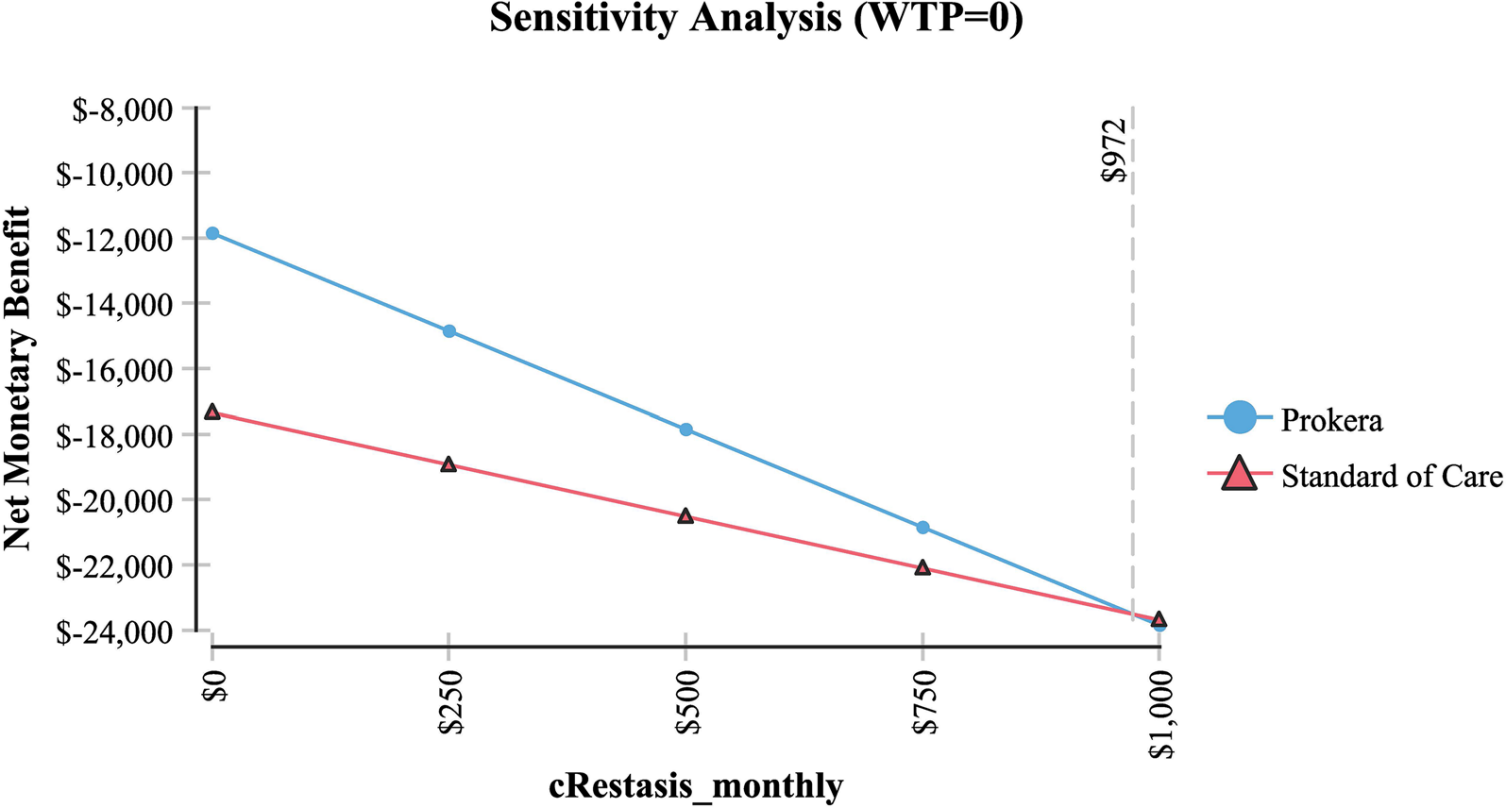
Sensitivity analysis showing that if the costs of Cyclosporine A (Restasis) are > 972/more then it is the less expensive option for treatment

**Fig. 6:**
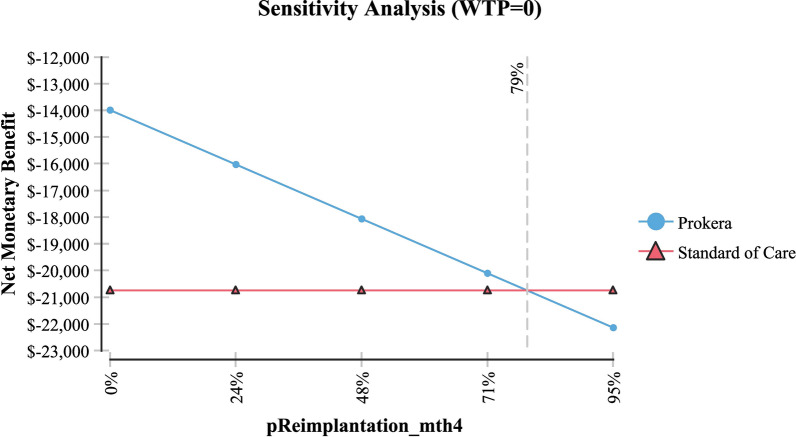
Sensitivity analysis showing that if amniotic membrane needs to be reimplanted >79% of the time at month 4, then Cyclosporine A (Restasis) is the less expensive option

**Fig. 7:**
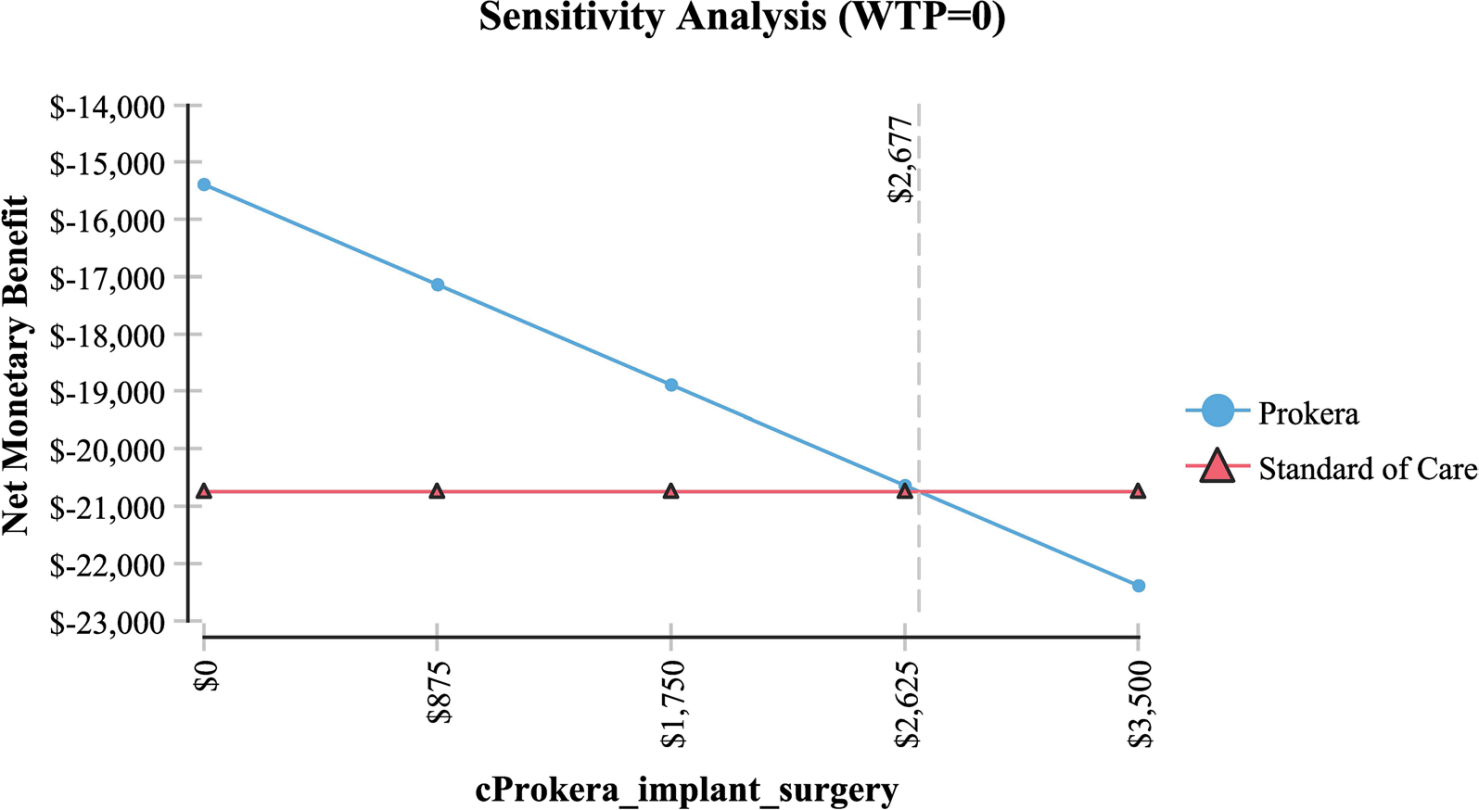
Sensitivity analysis showing that if the cost of the amniotic membrance surgical implantation is >$2,677 then Cyclosporine A (Restasis) becomes the less expensive option

**Fig. 8:**
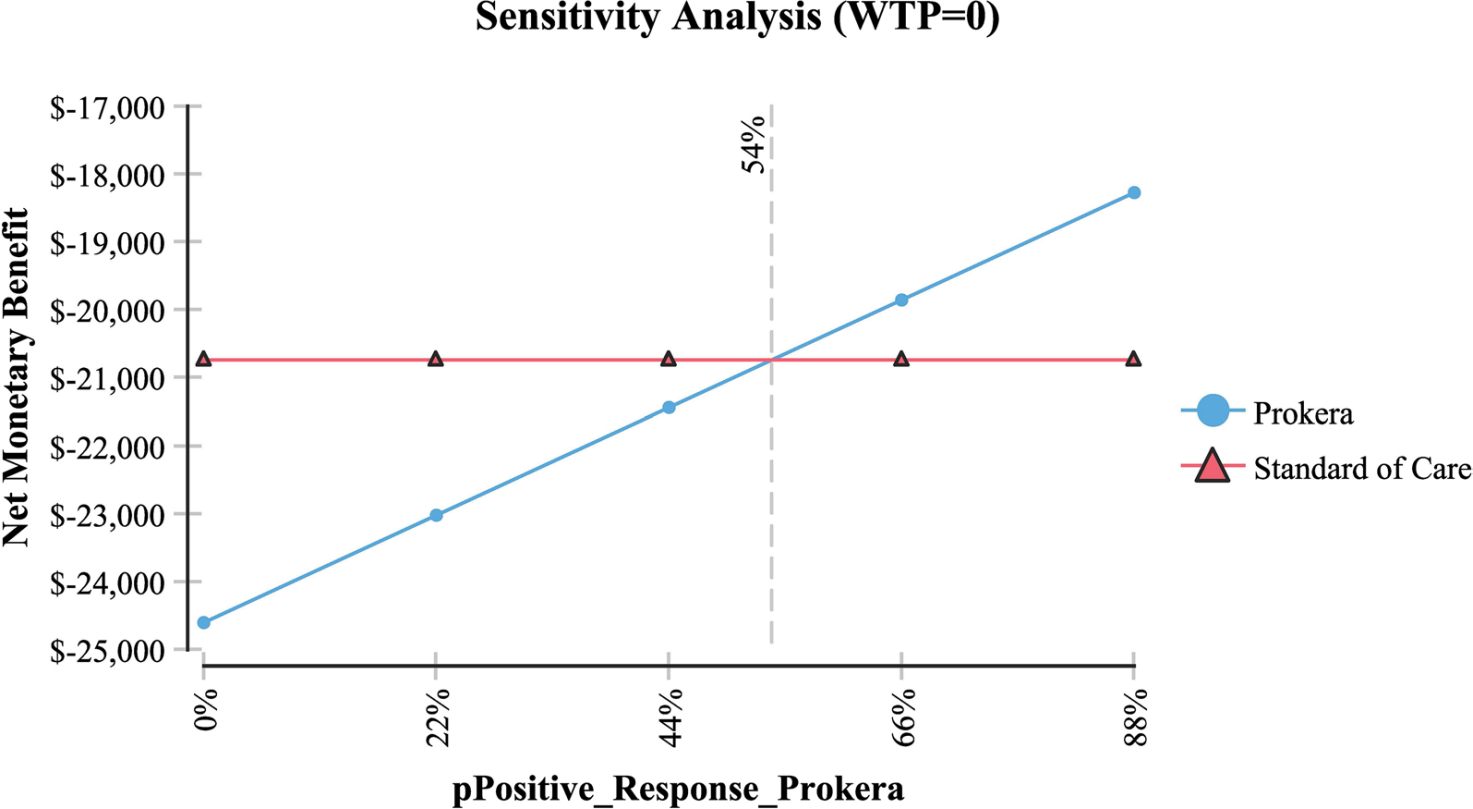
Sensitivity analysis showing that if the probability of a positive clinical response to Prokera is <54% then Cyclosporine A (Restasis) becomes the less expensive option

**Fig. 9:**
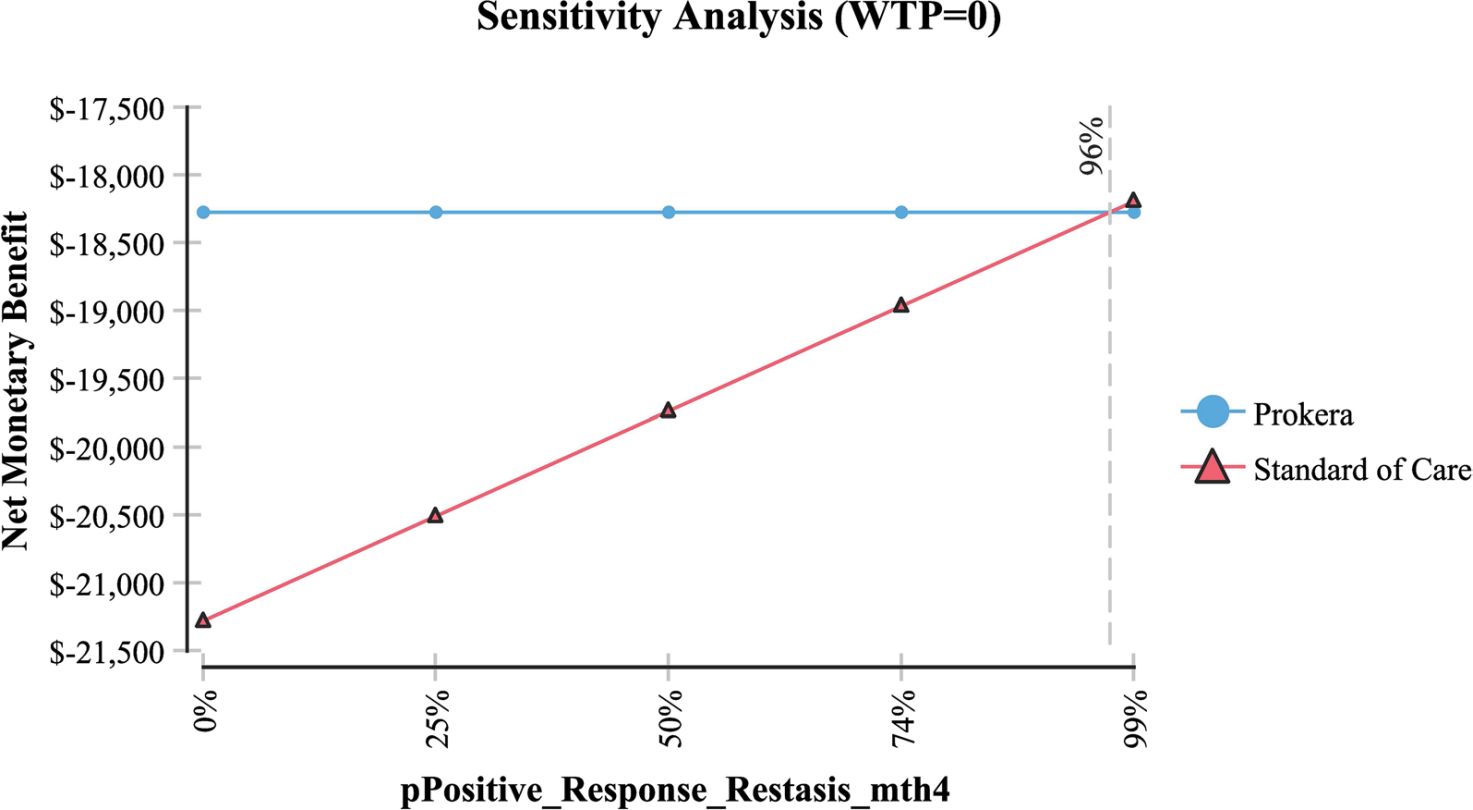
Sensitivity analysis showing that if the probability of a positive clinicial response to Cyclosporine A (Restasis) is >96% at month 4, then Cyclosporine A (Restasis) becomes the less expensive option

**Fig. 10:**
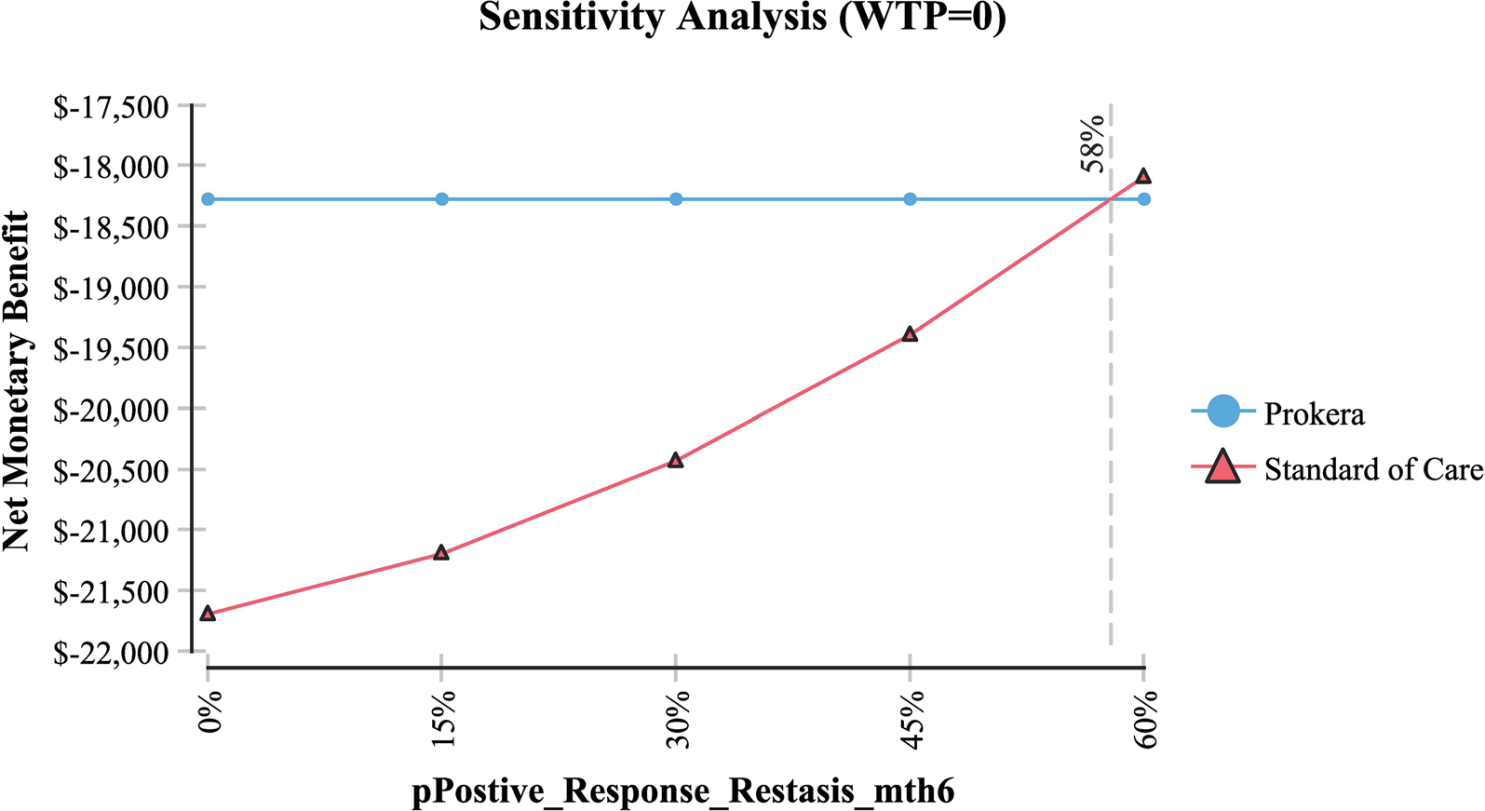
Sensitivity analysis showing that if the probability of a positive clinical response to Cyclosporine A (Restasis) is >58% at month 6, then Cyclosproine A (Restasis) becomes the less expensive option

**Fig. 11:**
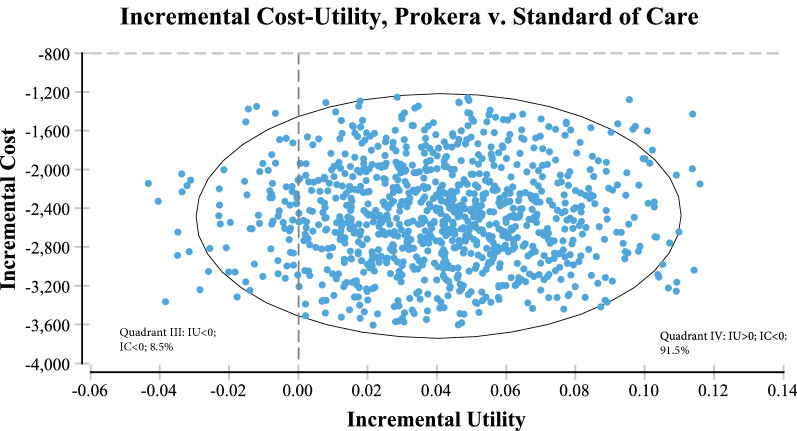
Incremental cost utility scatterplot demonstrating that >91% of the time, amniotic membrane is less costly and provides for improved outcomes/utility vs. Cyclosporine A (Restasis)

Monte Carlo simulation demonstrated that AM was the less costly, most effective alternative 91.5% of the time (Fig. 11). In other words, when all costs were evaluated (direct + indirect), AM dominated cyclosporine in providing cost utility (least costly with better outcome) the vast majority of the time. When examining only direct costs, topical cyclosporine was the least expensive option over a one year timeframe: $4112 vs. $10,300 with the same utilities. The incremental cost utility direct ratio (ICUR) using only direct costs resulted in an ICER for one year of [($10,300-$4112)/(0.78–0.74)] = $154,700 per quality adjusted life year (QALY).

## Discussion

The current analysis demonstrates that the use of AM + topical medications provides for cost utility vs. cyclosporine + topical medications in treating patients with moderate to severe DED. The main driver of cost savings comes from a lower likelihood of missed work days/lower work productivity with AM and; based on a higher likelihood that symptomatology is reduced with AM (i.e. indirect costs). The societal perspective in examining costs is important as sight affects a person’s QoL in a myriad of ways, including: driving, walking, working, and reading. Additionally, the psychological burden increases as vision decreases, along social aspects such as withdrawal from personal interactions due to impaired vision. The components of a good QoL may differ between people, but having visual acuity is typically a high priority for many people [23]. Thus it is important to reflect the effects of DED to society at large.

In order for cyclosporine A + topical medications to be the less expensive option, AM procedures need to significantly more expensive than they are currently; worker productivity needs to be significantly improved (i.e. societal outcomes with cyclosporine need to be significantly improved as it affects productivity); a very high rate of re-implantation of AM needs to occur every 4 months and; poorer outcomes with AM need to occur. As it relates to outcomes with AM, the clinical results as found in Table 1 demonstrate a very favorable outcome profile over 6–9 months; albeit with a limited number of patients [10–13]. With cyclosporine A, the clinical results as identified in the FDA phase III trial demonstrated a modest outcome improvement [20]. Based on this analysis, these outcomes would need to be a > 96% improvement at month 4 and a > 58% improvement at month 6 in order for cyclosporine A to be the less expensive option, an unlikely event considering the modest improvement identified in the trial results [20]. Further, considering the reimbursement for an AM implant (CPT 65778—placement of amniotic membrane on the ocular surface without sutures) is $1445; its reimbursement would need to be 85% higher (i.e. $2677) in order for cyclosporine A to be the less expensive option, again an unlikely event.

Prior studies have examined the cost-effectiveness of cyclosporine vs. sham [17]. This study demonstrated an ICUR of $34,953 per QALY for cyclosporine. The current study comparing AM + topical medications vs. cyclosporine A + topical medications demonstrated that in the vast majority of cases AM + topical medications dominated cyclosporine A + topical medications (i.e. was less costly and provided better outcomes). The inference drawn in this study, from a societal perspective, is that AM + topical medications should likely be a preferred therapy.

If one were to examine ICUR (using only direct costs), it was found that the IUER was $154,700/QALY. This ICUR reflected very aggressive therapy in that topical medications were not discontinued even if AM improved outcomes (e.g. use of AM resulted in an asymptomatic patient). If topical medications were discontinued in patients based on patients being asymptomatic with AM, the ICUR would be $79,475/QALY. Further, small improvements in utilities resulted in large improvements in outcome (e.g. going from moderate DED to asymptomatic outcome resulted in only a 0.04 utility improvement). Based on the data used for utilities [2], these small utility improvements were likely not reflective of the larger impact they had on clinical outcomes. The utility score improvements used in the ICER analysis for direct costs may thus have inflated the cost per QALY. However, despite this, using cost-utility upper limits of $100,000/QALY, AM ± topical medications at a $79,475/QALY (based on adjusting medications on symptomatology) might be considered cost effective when examining direct costs only [24].

One of the more interesting outcomes not addressed in this short term analysis is the potential effect of AM on corneal nerve regeneration [11] in DED and its effect on longer term sight/outcomes. As mentioned, neurosensory dysfunction, is not routinely tested for in DED [5]. If the inflammatory cascade of DED is not mitigated, changes can occur over time in the central nervous system (CNS) – and result in sight loss and pain hypersensitivity [5]. This pain is frequently associated with anxiety and depression [5]. Due to a rich supply of neurotrophic factors (e.g. nerve growth factors), AM has demonstrated a promotion of corneal nerve regeneration which result in a more lasting effect in treating DED [11]. Thus one of the added benefits of using AM to treat DED, is not only mitigating the inflammatory cascade, preventing further damage, but potentially in repairing damage already done [11]. Cyclosporine A only affects the inflammatory cascade to help reduce disease progression [25]. Additionally there are barriers to patient tolerance and acceptance of cyclosporine A therapy including burning, ocular stinging and conjunctival hyperemia [26]. To lessen these issues and make treatment more tolerable, clinicians have included topical corticosteroids as part of the cyclosporine therapy [27]. However, long term use of topical corticosteroids are associated with steroid-induced glaucoma, cataract formation, delayed wound healing, and increased susceptibility to infection [28].

## Limitations

This analysis assumed topical medications would be continued throughout the year, no matter the condition of the patients. As mentioned above, if the topical medications were discontinued, most especially in the AM + topical medication arm, direct costs in the AM arm would be reduced by approximately $3,000 per year. Further, it was assumed a longer term use of topical corticosteroids – upwards of one year was used in the analysis. Studies on the longer term use of topical corticosteroids are unclear on the risk of increased intra-ocular pressure, infection, or cataract development in patients with DED [29–32].

Patients who were entered into the model were assumed not to be chronic DED cases. Thus the effects of DED chronicity on such issues as neurosensory dysfunction and associated costs and sequelae were not captured in this analysis. Further, the short term nature (1 year of the analysis) did not reflect the longer term sequelae costs of such complications as neurosensory dysfunction.

Findings from a relatively small sample size of AM studies (n = 120; 4 studies) were used in the analysis. These studies did however demonstrate a consistency of finding – a positive clinical effect of AM on DED.

The costs for treating anxiety and pain were not evaluated in the cyclosporine A treatment arm nor were the longer term effects (costs and outcomes) of AM.

Since data was only available for a one year’s timeframe, the analysis of costs and outcomes was not extended beyond this timeframe.

Lastly, costs used in the analysis were derived from Medicare and reflected average purchase prices of medications and national average Medicare reimbursement rates. Based on Medicare payment rates to providers, efficient providers have a slightly negative margin [33] (reimbursement less costs). Therefore as a proxy for costs, Medicare payment rates appear to be close to costs.

## Supplementary information

**Additional file 1. **CHEERS checklist.

**Additional file 2. **PRISMA diagram.

**Additional file 3: **Variables and distributions.

**Additional file 4: **Decision tree with calculations

## Data Availability

All data generated or analyzed during the study are included in the published article and its supplementary files.

## Abbreviations

AM: Amniotic membrane
CPT: Current Procedural Terminology
DED: Dry Eye Disease
FDA: Food and Drug Administration
ICUR: Incremental Cost Utility Ratio
NADAC: National Average Drug Acquisition Cost
PRISMA: Preferred Reporting Items for Systematic Reviews and Meta-Analyses
QALY: Quality Adjusted Life Year
QoL: Quality of Life
TFOS: Tear, Film and Ocular Surface Society
TTO: Time Tradeoff
