# Supplementary material for: Cost utility analysis of cryopreserved amniotic membrane versus topical cyclosporine for the treatment of moderate to severe dry eye syndrome
Source: Cost Eff Resour Alloc. 2020 Dec 1;18:56. doi: 10.1186/s12962-020-00252-6 (PMC7709448; doi:10.1186/s12962-020-00252-6)
Supplement: Supplementary file 2 — Additional file 2. PRISMA diagram. [file 12962_2020_252_MOESM2_ESM.doc]

**Appendix 2: PRISMA 2009 Flow Diagram**

**Screening**

**Included**

**Eligibility**

**Identification**

Records identified through database searching including: PubMed, manufacturer info sent, manufacturer websites
(n = 55 )

Additional records identified through other sources –i.e. hand searches
(n = 2 )

Records after duplicates removed
(n = 53)

Records screened – i.e. abstracts reviewed
(n = 10 )

Records excluded
(n = 1 )

Full-text articles assessed for eligibility
(n = 9 )

Full-text articles excluded, with reasons
(n = 4 )

Studies included in qualitative synthesis
(n = 5 )

Studies included in quantitative synthesis

(n = 4 )

Pubmed search on 9/3/19 using the following search terms: ((cryopreserved) AND amniotic) AND membrane) AND ophthalmology) AND control*) AND trial

Product insert for Restasis identified 4 randomized controlled trials of which one RCT was used in the cost effectiveness analysis
