# Supplementary material for: Cost utility analysis of cryopreserved amniotic membrane versus topical cyclosporine for the treatment of moderate to severe dry eye syndrome
Source: Cost Eff Resour Alloc. 2020 Dec 1;18:56. doi: 10.1186/s12962-020-00252-6 (PMC7709448; doi:10.1186/s12962-020-00252-6)
Supplement: Supplementary file 3 — Additional file 3: Variables and distributions. [file 12962_2020_252_MOESM3_ESM.docx]

Appendix 3: Variables used in model

| **NAME** | **DESCRIPTION** | **FORMULA** | **VALUE** | **COMMENT** |
| --- | --- | --- | --- | --- |
| cArtificial_tears | Cost per month of artificial tears - Refresh plus | $18.00 | $18.00 | NADAC |
| cDurezol | Cost of one bottle of Durezol; 5mL - thirty day supply; NADAC pricing $35.80 | $35.80 | $35.80 | NADAC |
| cFollowup_care | Cost follow on care ophthalmic patient CPT 92012 | $89.74 | $89.74 | National aver. Medicare reimbursement 2019 |
| cIndirect_costs_productivity_loss_day | Indirect costs - productivity loss due to DED affecting performance. | $225.00 | $225.00 | Yu J, et al. The economic burden of dry eye disease in the US: A decision tree analysis. Cornea. 2011;30(4):379-387. Average hourly wage is $28.11 (BLS, August 2019). Equivalent lost works days due to moderate dry eye 95. Therefore: 95 X 8 X $28.11 = $21,364 |
| cOphthalmic_exam | Medicare reimbursement for CPT 92004 - comprehensive exam new patient; nonfacility | $153.53 | $153.53 | National aver. Medicare reimbursement 2019 |
| cProkera_implant_surgery | Cost Prokera implant plus surgical costs non-facility CPT 65778 | $1,444.45 | $1,444.45 | CPT 65778 reimbursed at $1,444.45. Includes cost of implant in supply cost for non-facility setting, Medicare 2019 rates |
| cPunctalSurgery | Cost closure lacrimal punctum - CPT 68760; Medicare 2019 rate | $213.35 | $213.35 | National aver. Medicare reimbursement 2019 |
| cRestasis_monthly | Cost monthly supply of Restasis; NADAC pricing @ $8.93/vial | $535.80 | $535.80 | 60 vials in a 30 days supply; NADAC |
| cTearDuctSurg | Cost close tear duct surgery CPT 68761; Medicare rate 2019 | $152.09 | $152.09 | National aver. Medicare reimbursement 2019 |
| number_days_lost_DED | Average number of days lost due to DED | Days_Lost_ModDED | 94.87 | Yu J, et al. The economic burden of dry eye disease in the US: A decision tree analysis. Cornea. 2011;30(4):379-387. Average hourly wage is $28.11 (BLS, August 2019). Equivalent lost works days due to moderate dry eye 95. Therefore: 95 X 8 X $28.11 = $21,364 |
| pCondition_Worsens_Restasis | Probability that patients DED worsens | 0.04 | 0.04 | Derived from Restasis NDA 21-023 study. 4% of patients in study, condition worsened at 6 months |
| pPositive_Response_Prokera | Probability improved ocular surface with Prokera. | 0.88 | 0.88 | McDonald MD, et al. Treatment outcomes in the dry eye amniotic membrane (DREAM) study. Clin Ophthal 2018;12:677-681. |
| pPositive_Response_Restasis_mth4 | Probability of a positive response (>50% global response) Restasis - month 4. | 0.173 | 0.17 | CDER application number 21-023 at month 4. 26 out of 150 patients with marked response and able to work at normal productivity |
| pPostive_Response_Restasis_mth6 | Probability of a positive response (>50% global response) with Restasis month 6. | 0.245 | 0.25 | Brown MM et al. Value-based medicine, comparative effectiveness, and cost-effectiveness analysis of topical cyclosporine for the treatment of dry eye syndrome. Arch Ophthal. 2009;125(2):146-1520.245 |
| pReimplantation_mth4 | Probability Prokera reimplantation at mth 4 | 0.5 | 0.50 | Estimate |
| pReimplantation_mth8 | Probability of Prokera reimplanation at month 8 | 0.5 | 0.50 | Estimate |
| uGain_Prokera | Average utility gain with use of Prokera - going from moderate to asymptomatic | 0.04 | 0.04 | Source of data: Schiffman RM, et al. Utility assessment among patient with dry eye disease. Ophthal. 2003;110:1412-1419. Patient goes from moderate DED 0.82 utility to asymptomatic 0.86 utility; increase of 0.04. |
| uGain_Restasis | Average utility gain with use of Restasis. Includes disutility of adverse events from Restasis. | 0.0534 | 0.05 | Source: Brown MM, et al. Value-based medicine, comparative effectiveness, and cost-effectiveness analysis of topical cyclosporine for the treatment of dry eye syndrome. Arch Ophthal. 2009;127(2):146-152. |
| uLoss_per_status | Utility loss going from moderate to severe dry eye | Utility_Loss_per_status | 0.07 | Schiffman RM, et al. Utility assessment among patient with dry eye disease. Ophthal. 2003;110:1412-1419. |
| uModerateDED | Clinical utility dry eye disease | ModerateDED | 0.78 | Schiffman RM, et al. Utility assessment among patient with dry eye disease. Ophthal. 2003;110:1412-1419. |

Appendix 3: Distributions used in model

| **Type** | **Name** | **Description** | **Param 1** | **Param 2** | **Param 3** |
| --- | --- | --- | --- | --- | --- |
| Triangular | Days_Lost_ModDED | Number of days lost moderate dry eye disease | 81.1 | 94.9 | 108.6 |
| Normal | ModerateDED | Clinical utility of moderate dry eye | 0.78 | 0.19 |  |
| Normal | Utility_Loss_per_status | Utility loss going from moderate to severe dry eye | 0.07 | 0.07 |  |
