# Supplementary material for: Cost utility analysis of cryopreserved amniotic membrane versus topical cyclosporine for the treatment of moderate to severe dry eye syndrome
Source: Cost Eff Resour Alloc. 2020 Dec 1;18:56. doi: 10.1186/s12962-020-00252-6 (PMC7709448; doi:10.1186/s12962-020-00252-6)
Supplement: Supplementary file 4 — Additional file 4: Decision tree with calculations [file 12962_2020_252_MOESM4_ESM.docx]

Appendix 4: Decision tree with calculations

| **Therapy** | **Month 1-4** | **Month 5-8** | **Month 9-12** | **Cost** | **Probability** | **Cost*Prob** | **Calculation** |
| --- | --- | --- | --- | --- | --- | --- | --- |
| Prokera | Prokera implant | Prokera implant | Prokera implant | $33,312 | 3.0% | $999 | cOphthalmic_exam+cProkera_implant_surgery+cFollowup_care+cProkera_implant_surgery+cFollowup_care+cProkera_implant_surgery+cIndirect_costs_productivity_loss_day*number_days_lost_DED*8/12+cReStasis_monthly*12+cDurezol*12+cArtificial_tears*12 |
| Prokera | Prokera implant | Prokera implant | Stasis/improve | $24,590 | 3.0% | $738 | cOphthalmic_exam+cProkera_implant_surgery+cFollowup_care+cProkera_implant_surgery+cFollowup_care+cReStasis_monthly*12+cDurezol*12+cArtificial_tears*12+cIndirect_costs_productivity_loss_day*number_days_lost_DED*4/12 |
| Prokera | Prokera implant | Stasis/improve | Prokera implant | $24,527 | 3.0% | $736 | cOphthalmic_exam+cProkera_implant_surgery+cFollowup_care+cFollowup_care+cProkera_implant_surgery+cReStasis_monthly*12+cDurezol*12+cArtificial_tears*12+cIndirect_costs_productivity_loss_day*number_days_lost_DED*4/12 |
| Prokera | Prokera implant | Stasis/improve | Stasis/improve | $15,968 | 3.0% | $479 | cOphthalmic_exam+cProkera_implant_surgery+cFollowup_care+cFollowup_care+cReStasis_monthly*12+cDurezol*12+cArtificial_tears*12 |
| Prokera | Prokera implant | Prokera implant | Prokera implant | $25,972 | 22.0% | $5,714 | cOphthalmic_exam+cFollowup_care+cFollowup_care+cReStasis_monthly*12+cDurezol*12+cArtificial_tears*12 |
| Prokera | Prokera implant | Prokera implant | Stasis/improve | $17,412 | 22.0% | $3,831 | cOphthalmic_exam+cFollowup_care+cFollowup_care+cPunctalSurgery*pCondition_Worsens_ReStasis+cReStasis_monthly*12+cDurezol*12+cArtificial_tears*12+cIndirect_costs_productivity_loss_day*number_days_lost_DED*4/12 |
| Prokera | Prokera implant | Stasis/improve | Prokera implant | $17,412 | 22.0% | $3,831 | cOphthalmic_exam+cFollowup_care+cPunctalSurgery*pCondition_Worsens_ReStasis+cFollowup_care+cReStasis_monthly*12+cDurezol*12+cArtificial_tears*12+cIndirect_costs_productivity_loss_day*number_days_lost_DED*4/12 |
| Prokera | Prokera implant | Stasis/improve | Stasis/improve | $8,853 | 22.0% | $1,948 | cOphthalmic_exam+cFollowup_care+cPunctalSurgery*pCondition_Worsens_ReStasis+cFollowup_care+cTearDuctSurg*pCondition_Worsens_ReStasis+cReStasis_monthly*12+cDurezol*12+cArtificial_tears*12+cIndirect_costs_productivity_loss_day*number_days_lost_DED*8/12 |
| **Totals** |  |  |  |  | **100.0%** | **$18,275** |  |
|  |  |  |  |  |  |  |  |
| ReStasis | Improvement | Improvement | Improvement | $7,408 | 1.0% | $74 | cOphthalmic_exam+cFollowup_care+cFollowup_care+cReStasis_monthly*12+cDurezol*12+cArtificial_tears*12 |
| ReStasis | Improvement | Improvement | Punctal surgery | $14,532 | 3.2% | $465 | cOphthalmic_exam+cFollowup_care+cFollowup_care+cPunctalSurgery*pCondition_Worsens_ReStasis+cReStasis_monthly*12+cDurezol*12+cArtificial_tears*12+cIndirect_costs_productivity_loss_day*number_days_lost_DED*4/12 |
| ReStasis | Improvement | Punctal surgery | Improvement | $14,532 | 3.2% | $465 | cOphthalmic_exam+cFollowup_care+cPunctalSurgery*pCondition_Worsens_ReStasis+cFollowup_care+cReStasis_monthly*12+cDurezol*12+cArtificial_tears*12+cIndirect_costs_productivity_loss_day*number_days_lost_DED*4/12 |
| ReStasis | Improvement | Punctal surgery | Tear duct surgery | $21,653 | 9.9% | $2,144 | cOphthalmic_exam+cFollowup_care+cPunctalSurgery*pCondition_Worsens_ReStasis+cFollowup_care+cTearDuctSurg*pCondition_Worsens_ReStasis+cReStasis_monthly*12+cDurezol*12+cArtificial_tears*12+cIndirect_costs_productivity_loss_day*number_days_lost_DED*8/12 |
| ReStasis | Punctal surgery | Improvement | Improvement | $14,532 | 5.0% | $727 | cOphthalmic_exam+cPunctalSurgery*pCondition_Worsens_ReStasis+cFollowup_care+cFollowup_care+cReStasis_monthly*12+cDurezol*12+cArtificial_tears*12+cIndirect_costs_productivity_loss_day*number_days_lost_DED*4/12 |
| ReStasis | Punctal surgery | Improvement | Tear duct surgery | $21,653 | 15.3% | $3,313 | cOphthalmic_exam+cPunctalSurgery*pCondition_Worsens_ReStasis+cFollowup_care+cFollowup_care+cTearDuctSurg*pCondition_Worsens_ReStasis+cReStasis_monthly*12+cDurezol*12+cArtificial_tears*12+cIndirect_costs_productivity_loss_day*number_days_lost_DED*8/12 |
| ReStasis | Punctal surgery | Tear duct surgery | Improvement | $21,799 | 15.3% | $3,335 | cOphthalmic_exam+cPunctalSurgery*pCondition_Worsens_ReStasis+cFollowup_care+cTearDuctSurg+cFollowup_care+cReStasis_monthly*12+cDurezol*12+cArtificial_tears*12+cIndirect_costs_productivity_loss_day*number_days_lost_DED*8/12 |
| ReStasis | Punctal surgery | Tear duct surgery | Tear duct surgery | $21,699 | 47.1% | $10,220 | cOphthalmic_exam+cPunctalSurgery*pCondition_Worsens_ReStasis+cFollowup_care+cTearDuctSurg*pCondition_Worsens_ReStasis+cFollowup_care+cTearDuctSurg*pCondition_Worsens_ReStasis+cIndirect_costs_productivity_loss_day*number_days_lost_DED |
| **Totals** |  |  |  |  | **100.0%** | **$20,743** |  |
